# Addressing change in physiotherapy education in South Africa

**DOI:** 10.4102/sajp.v74i1.431

**Published:** 2018-03-27

**Authors:** Seyi L. Amosun, Soraya Maart, Niri Naidoo

**Affiliations:** 1 Department of Health and Rehabilitation Sciences, University of Cape Town, South Africa

## Abstract

**Background:**

Recent demands for the decolonisation of curriculum in South Africa present challenges to students, academics and other stakeholders. This resulted in tensions in tertiary institutions, cumulating in student-led protests. The authors hypothesised that the lack of shared understanding of what this unexplored process may entail contributed to the dilemma.

**Objective:**

The aim of this opinion article is to highlight some of the possible contributors to the uncertainties in addressing this critical issue, especially as it relates to the demands for change in physiotherapy education.

**Method:**

To formulate our opinion, the authors reviewed literature relating to transformation in education in South Africa generally, and physiotherapy education specifically.

**Results:**

While there is an opportunity to address the demand for change in physiotherapy education in South Africa, there is the possibility that the use of words, such as transformation, decolonisation and decoloniality, present different connotations to students and academics.

**Conclusion:**

It is of vital importance to create formal discourse which includes students, academics and other stakeholders that will facilitate shared understanding about what the previously unexplored and unmapped processes of engagement entail. The change process in physiotherapy education is envisaged to be a partnership between students and academic staff having common understanding about the processes and responsibilities, and must be addressed comprehensively.

**Clinical implications:**

Aligning the change process in physiotherapy education with the decolonisation agenda will strengthen the South African health care system by ensuring that physiotherapy students are adequately prepared to provide service to patients within a context that acknowledges the uniqueness of South African communities.

## Background

The national students’ protests in 2015 kick-started the demand for the decolonisation of curricula in higher education in South Africa (Davids & Waghid 2016). This created anxious moments for both staff and students and invoked strong emotions and responses. We hypothesise that the major causes of the anxiety were the lack of understanding of what the demands entailed and the uncharted processes to address the demands. Davids and Waghid (2016) reported that the most recent ‘fees must fall’ protests have involved students from both historically advantaged and disadvantaged universities, and sparked a reminder of post-apartheid South Africa’s embedded inequalities. Linking these developments to tertiary education presents a dilemma to many. A significant outcome of the protests is the opportunity for academic staff and students to engage on how to bring about the changes demanded. The aim of this opinion article therefore is to highlight some of the possible contributors to the dilemma, especially as it relates to the demands for change in physiotherapy education, in order to seize the opportunity to review the curriculum as part of the process for meaningful change. It is important to highlight from the onset that while the authors are not ‘experts’ in curriculum change, they are willing participants and learners while going through the uncharted processes.

In an attempt to increase the numbers of healthcare professionals globally, the Lancet’s Global Independent Commission report on Education of Health Professionals for the 21st century, advocated that there should be changes in the educational preparation of healthcare professionals for a world that is experiencing a rapid transition from acute diseases to chronic health problems which persist throughout the lifespan (Frenk et al. 2010). The commission noted the inequities in health which the current training strategies seem not to address adequately. Two of the recommendations of the commission relate to ensuring that the training programmes are transformative in producing change agents, and promoting inter-professional and trans-professional education. The World Health Organization (2013) also provided guiding principles for transforming and scaling up health professionals’ education and training, to ensure that graduates become change agents who are socially accountable.

The World Confederation of Physical Therapy (WCPT) developed five international guidelines for entry-level physiotherapy education (Moffat 2012), and three key elements were identified in designing the curriculum for undergraduate physiotherapy education, namely the content or knowledge base, students’ learning process and the sociocultural context in which physiotherapy is experienced and practised (Lindquist, Engardt & Richardson 2010; McMenamin et al. 2014; Van Heerden 2013). The history of physiotherapy education globally portrays the influence of colonial masters on physiotherapy practice and education in Africa (Moffat 2012). For example, in South Africa, the early origins of physiotherapy practice began in hospital-based settings to address the rehabilitation needs resulting from World War I causalities and for individuals impacted by disease epidemics, like poliomyelitis. Consequently, physiotherapy education was introduced. Ramklass (2009) reported that until 1994, physiotherapy education aligned with the expectations of the South African health system. However, post-apartheid, the curricula have remained relatively static, and little has changed in making the programmes more inclusive (Amosun et al. 2012a, 2012b). The call for transformative health professional education is therefore critical, in terms of the relevance of the curriculum and access to services, for South Africa which is still experiencing a high burden of disease and inequities in health (Amosun et al. 2012a, 2012b; Mayosi & Benatar 2014).

## Power of words

The growing demand for change in higher education in South Africa has been linked to a particular discourse of transformation in the context of decolonisation and decoloniality. The application of these concepts remains a grey area in ongoing dialogues. In addition, there are uncertainties on who should undertake the processes involved in transformation, decolonisation or decoloniality. It is therefore crucial to develop shared understandings and ideas in these dialogues for change in physiotherapy education in South Africa. The intention of this opinion article therefore is to highlight how the lack of a collective understanding of these words may be contributing to the uncertainties generated in the dialogues.

We suggest that a starting point in the dialogue for change in physiotherapy education is an understanding of the definition of physiotherapy. The WCPT (2011) issued a policy statement describing physiotherapy based on the premise that functional movement is central to what it is to be healthy and would therefore encompass physical, psychological, emotional and social well-being. Physiotherapy is concerned with maximising quality of life and movement potential within the spheres of promotion, prevention, treatment or intervention, habilitation and rehabilitation. The services are provided to individuals and population groups in circumstances where movement and function are threatened by ageing, injury, pain, diseases, disorders or environmental factors. The goals are to develop, maintain and restore movement and functional ability throughout the lifespan.

Another point worth emphasising is an understanding of the context in which the dialogue is taking place. The Republic of South Africa is a multi-ethnic nation, with an increasing number of immigrants (Statistics South Africa 2015). Despite being classified as an upper middle-income country, South Africa has the highest level of inequality in the world, in which the poorest one-fifth of the population accounts for 2% of the country’s income and consumption, and the richest one-fifth accounts for 72% (Rowe & Moodley 2013). The country also experiences a quadruple burden of diseases, namely HIV and AIDS with a synergistic relationship with tuberculosis; maternal and child morbidity and mortality; non-communicable diseases mostly driven by risk factors related to lifestyle; and violence, injuries and trauma (Mayosi & Benatar 2014). The lives of South Africans have been impacted by many years of apartheid in the form of inequities in social and health care, among others. The government therefore developed the National Development Plan (NDP) 2030 as part of the transformation process to correct the inequities (Alloggio & Thomas 2013).

Transformation is one of the terms that have been used in the dialogues for change in physiotherapy education in South Africa. The *Oxford English Dictionary* defined ‘transformation’ as ‘a marked change in form, nature, or appearance’. As we reflected on this definition, some questions arose – Is this ‘future’ form, nature or appearance predetermined? If yes, by whom? If not, who determines when this change has taken place? Was the ‘future’ form, nature or appearance of the physiotherapy education programme in South Africa predetermined? If yes, who did? If not, who determines when the programme is transformed? Transformation is still often loosely defined in higher education in South Africa, emphasising mainly that transformation should ‘reflect the changes that are taking place in our society’ (Du Preez, Simmonds & Verhoef 2016:1). One of the major transformational challenges in education, including physiotherapy education, was the process of widening access into higher education to students from previously disadvantaged backgrounds (Amosun et al. 2012a, 2012b). However, this has not changed the face of physiotherapy students at some universities. This is further reflected in the membership of the South African Society of Physiotherapy. Appreciating the fact that the society is a non-profit organisation that is committed to equal opportunities and inclusivity in a country with the highest level of inequity in the world (Rowe & Moodley 2013), and that office-bearers are volunteers, participation may be limited to those who have the resources to volunteer.

Unfortunately, the transformation processes in physiotherapy education still present serious challenges to both the academics and students (Ige, Amosun & Hartman 2017). A major concern related to processes which were facilitated mainly by academics though making use of students’ data and the accompanying curriculum overload (Amosun et al. 2012a, 2012b). It is perceived that many of the goals for transformation of the higher education sector in South Africa are yet to be achieved (Du Preez et al. 2016), such as aligning the curriculum within the South African context, increasing access to previously disadvantaged students and increasing the number of demographically representative staff members. We hypothesise that this triggered the students’ protests in 2015, which gave rise to student-led dialogues on decolonisation of the curriculum, because of perceptions that very little has changed in higher education despite the widening of access.

The use of the word ‘decolonisation’ also presented its challenges. The *Oxford English Dictionary* defined colonisation as ‘the action or process of settling among and establishing control over the indigenous people of an area’. Similarly, decolonisation is described as ‘the withdrawal from its colonies of a colonial power; the acquisition of political or economic independence by such colonies’. As the colonisers of South Africa are predominantly Europeans, theoretically decolonisation could be construed as ‘undoing’ everything the colonisers (white people) have done. This certainly created much discomfort among many South African academics from all population groups (Davids & Waghid 2016). As authors, we believe that the perception that decolonising the curriculum is a deliberate replacement of knowledge created by one population group with that of another population group is totally misplaced. However, it is vitally important to decolonise the misconception of the superiority of Western Eurocentric knowledge in order to debunk the belief that this is the only viable knowledge when it comes to education (Higgs 2016).

Higher education in South Africa in the 21st century is expected to operate in both a postcolonial and a globalising context (Higgs 2016). It is the authors’ opinion that the curriculum in higher education in postcolonial South Africa is unfortunately still to a large extent confronted by the legacy of colonial education, which remained in place decades after political decolonisation. This raises the curiosity in identifying the postcolonial changes in physiotherapy education in South Africa. For example, how far have the recommendations on the Education of Health Professionals for the 21st century been incorporated into the physiotherapy education curriculum? This speaks of the epistemological nature of knowledge and how it relates to truth, belief and justification; furthermore, it also deals with the means of production of knowledge, as well as scepticism about different knowledge claims (Bientzle, Cress & Kimmerle 2014). Unfortunately, the Council of Higher Education (2013) concluded that fundamental changes to higher education curricula have not happened across South African higher education institutions for nearly a century, as existing curricula, including physiotherapy, still bear a close resemblance to colonial education. The changes made have been mostly cosmetic in response to some of the social and health issues, such as the invisible power structures which lie within Eurocentric ideologies.

The discontent of students expressed in the 2015 protests has offered opportunities to again engage with existing curricula in physiotherapy, but ‘reflecting the changes that are taking place in our society’ (Du Preez et al. 2016:1). The alternative is to continue to offer physiotherapy education under the legacy of colonial education, though realising that sustainable education requires an innovative approach to knowledge acquisition and learning. A major feature of colonial education is ‘the asymmetrical power imbalance between students and teachers’ (Keet, Sattarzadeh & Munene 2017:8). We hypothesise that this is where the issue of decoloniality arises, and it relates to both academic staff and students. For the purpose of this opinion article, we describe decoloniality as the ‘art of undoing colonial education’. A first step involves a process of self-understanding – ‘How we (staff and students) see ourselves doing academic work or living our academic lives’ (Keet et al. 2017:4). Physiotherapy students therefore have a choice to continue to be passively or be actively engaged in the acquisition of knowledge and learning. Academic staff also have the choice of either maintaining the status quo or, seizing the opportunities in the context of academic freedom and scholarly autonomy, being at the forefront of knowledge and curriculum renewals (Keet et al. 2017). Students being actively engaged in knowledge acquisition and learning will contribute to their development and growth, in preparation for becoming leaders of change in society. Academic staff will also be able to reflect on and challenge their own epistemology as academic developers.

## Moving forward

The process of change in physiotherapy education in South Africa is not a domain reserved exclusively for students or academic staff. The creation of formal dialogue spaces for students and academics will help to build common understanding in the use of these words by interrogating what these terms mean, and overcome the uncertainties. Students should also be actively involved in the fresh opportunity to review physiotherapy education curricula and should address the challenge of curricula overload and approaches to teaching, learning and assessment.

## Conclusions

The demand for change in physiotherapy education in South Africa should be a partnership between students and academic staff, having common understandings about the processes and responsibilities, and must be addressed comprehensively. The intention of the authors is not to prescribe but rather to highlight the limiting factors to change. It is for members of the physiotherapy profession and stakeholders to strive for a cohesive physiotherapy identity that is rooted within the African perspective, while acknowledging global perspectives.
